# Understanding Mobile Money Grievances from Tweets

**DOI:** 10.1145/3287098.3287123

**Published:** 2019-01-04

**Authors:** Kushal Shah, Shrirang Mare, Richard Anderson

**Affiliations:** University of Washington

**Keywords:** Mobile Financial Services, ICTD, Twitter, Financial Inclusion

## Abstract

Access to financial services through a mobile phone, known as Mobile Financial Services (MFS), creates an opportunity to expand the reach of financial services to the 1.7 billion unbanked adults worldwide. Nevertheless, MFS adoption has been inconsistent, which motivates a need to identify the challenges that MFS users confront in different countries. In this work, we explore the Twitter as a potential data source to understand such challenges. More broadly, we assess whether (and how) publicly available Twitter data can augment the findings of expensive, large-scale research studies on MFS barriers. Our Qualitative Content Analysis of 9,000 mobile money grievance tweets that were extracted from 54 MFS customer care twitter feeds across six countries reveals *service and access issues, incorrect transactions*, and *fraud* as three main challenges MFS users report on Twitter. We discuss the nuances around these challenges and the substantial differences between the common issues reported in different countries. Ultimately, we conclude that Twitter data can elucidate the challenges of MFS adoption and also that it can augment the results of other types of MFS studies.

**CCS CONCEPTS:**

• **Human-centered computing** *Human computer interaction (HCI)*; *Social media*; • **Applied computing** *Sociology*;

## 1 INTRODUCTION

Financial inclusion, in which people have access to savings, credit, remittance products, and financial institutions, is recognized as a component of lifting people out of poverty worldwide [1, 10, 34, 39]. Mobile Financial Services (MFS), where access to financial products is through a mobile phone, creates the opportunity for expanding the reach of financial institutions and creating new services which can serve more people [21, 37]. Kenya stands out as the notable success, where mobile money, led by M-Pesa, is used by 73% of the adult population. Thus, there is great interest by the development community in understanding the barriers to adoption of these services, both to increase adoption and to support financial inclusion.

Multiple studies seek to understand the barriers for adoption and use of MFS. Generally, these studies involve in-depth interviews with different stakeholders of financial services [19, 38], randomized controlled trials (RCTs) [4], large scale nationally representative surveys [14, 17], or longitudinal surveys [14, 21]. While all these types of studies may yield important insights about MFS, the methods are expensive and time-consuming. We explore an opportunity to augment the results of these studies by analyzing Twitter data. Twitter has become a common channel through which customers complain about a service and seek customer care support [2, 12, 20, 30]. Many mobile money service providers use customer care Twitter handles where users can report their complaints, ask questions, and seek help regarding the service. Thus, Twitter is potentially a rich dataset about issues reported by mobile money users, and in this paper, we investigate the use of this data for understanding the challenges faced by consumers in adopting MFS in different countries.

The goal of this study is to analyze Twitter data and highlight problems that MFS users in Ghana, India, Kenya, Pakistan, South Africa and Uganda tweet about, and how the corresponding customer service dialogue takes place. We randomly sample 1500 tweets from from all the six countries and then perform qualitative analysis to label the tweets. The tweets were labeled in multiple iterations, the first to identify whether a given tweet was related to MFS and the second to mark the MFS problem. We then condensed our labels into several higher level categories such as service error, transaction reversal, access error, fraud, etc.

We complement this qualitative analysis with other studies undertaken to understand MFS barriers. Our findings indicate that Twitter is a cost-effective and, surprisingly, rich data source to understand problems of MFS users. We find that service issues, where users report transaction delays, and access issues, where they are unable to login, are the most common problems. We then discuss the strengths and weaknesses of our analysis, and provide recommendations to make the findings more robust using cross-cultural analysis and quantitative methods. Finally, we present ways to address the challenges that our analysis highlights.

## 2 RELATED WORK

### 2.1 MFS for Financial Inclusion

Research on MFS ranges from studies that explore financial systems and money to those that directly evaluate the technologies that implement mobile money. Kumar [22] explored that rapidity of transactions, flexibility of bargaining, and complexity of change making that have ramifications for mobile systems. Pal [31] looked at shop-keeper payments in India. O’Neill [29] argues that the means of payments is a component of a larger social process. Blumenstock [4] examined that government payments did not go to the beneficiaries. Interview and survey based studies have drawn attention to a wide range of barriers to MFS adoption. Yu [40] identified high transaction costs, technological limitations, and limited need as issues. Ibtasam [19] considers gender and societal barriers as fundamental obstacles. Ghosh [16] draws attention to consumer lack of understanding of financial concepts. Medhi [27] identified agent proximity, transaction costs, and perceived reliability in a multi-country study. Technical work in computing has sought to address specific MFS challenges. Medhi [26] studied how to make mobile money systems more accessible to low-literate. Ibtasam [18] explored the usability and learnability of smartphone mobile wallet applications. Vulnerability of mobile money through “thin-sim” attacks is explored by Phipps [32]. The problem of security of mobile apps is evaluated by Reaves [33], and later by Castle [5].

### 2.2 Grievance Redressal

Mobile technologies have created opportunities for reporting complaints across a wide range of domains. CGNET Swara [24, 28] was developing a mechanism for remote, disconnected users to participate. Marathe [25] studied the scaling of complaint redress. Chakrabory [6, 7] has studied the impact of complaints and redress in jobs programs. Gaut [15] looks at automatic methods of complaint classification for appropriate routing. A common theme in works studying complaints is the interplay between supporting technology, human responses, and organizational incentives for resolution.

### 2.3 Twitter studies

Twitter has inspired a breadth of academic research on areas ranging from understanding misinformation [3] to extracting sentiments using automated classification systems [41]. Dunphy [11] sampled 698 tweets with the hashtag “#password” and then performed Qualitative Content Analysis to identify themes and then label tweets into higher-level categories. Kwizera [23] developed an automated chatbot system for the Kenyan Customer Service Market. Fichet [13] examined how back and forth conversations on Twitter aided in crisis relief.

## 3 MFS CHALLENGES

**Figure 1:**
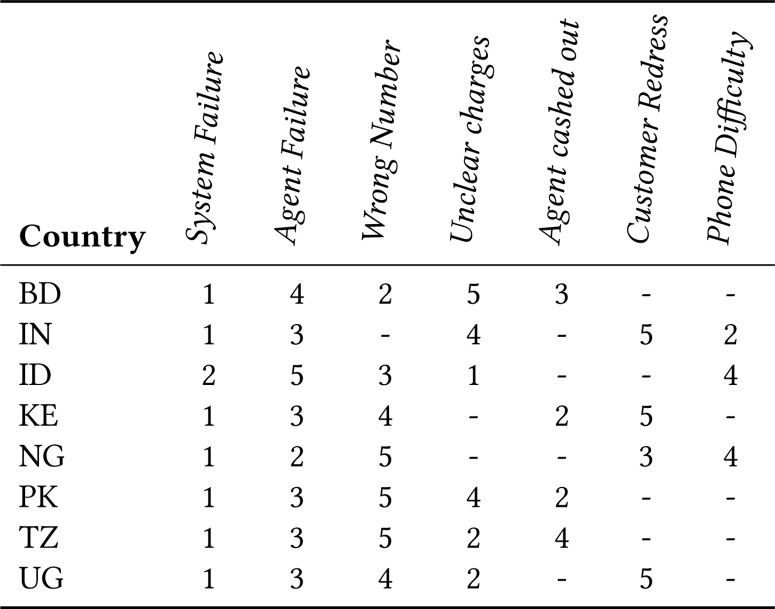
Top 5 Mobile Money Challenges from FII

One of the most significant efforts to understand MSF challenges has been the Financial Inclusion Insights (FII) Program by Intermedia [14]. This includes large scale surveys which have been done annually across Pakistan, India, Tanzania, Uganda, Kenya, Nigeria, Indonesia and Bangladesh to understand financial readiness. The survey asks about mobile money usage and the problems that users face when using mobile money services. We give the rank order lists of challenges faced by mobile money users in Figure 1. In Section 7 we compare the challenges identified through Twitter with the FII findings of the five countries that overlap from our study and the FII surveys.

## 4 METHODOLOGY

### 4.1 Twitter Data Collection and Filtering

We chose Bangladesh, Ghana, India, Kenya, Nigeria, Pakistan, Tanzania, South Africa, Senegal and Uganda as the initial countries of focus. A Python script ran continuously to collect data from September 2016 until June 2018 and collected 569, 301 tweets.

**Figure 2:**
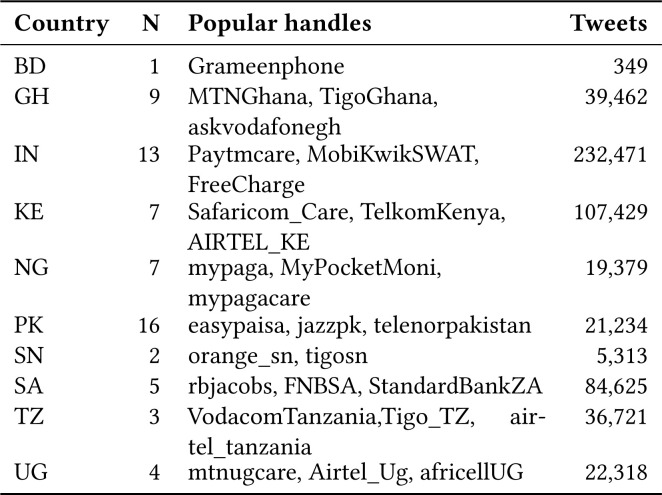
Summary of MFS Twitter handles and data parsed; N is the number of handles parsed for a country.

The typical Twitter customer care exchange begins with users sharing their problems by mentioning the Twitter handle of the MFS operator. A customer service representative responds and then a series of back and forth messages take place. We refer to such tweets where the MFS providers responds as *actionable* tweets. We decided to consider only English tweets which were actionable and hence decided to consider only Ghana, India, Kenya, Pakistan, South Africa and Uganda for our analysis.

### 4.2 Qualitative Content Analysis

From a total of 18,302 actionable tweets across the six countries, we randomly sampled 1500 tweets per country and labeled tweets which were MFS related, and then labeled them into higher and lower-level categories. In Figure 6, we show the counts of total MFS related tweets from the sample. For example, in the following tweet: (*“sent money to wrong number and I didn’t know”*), we labelled this tweet with the reversal and incorrect transaction as higher and lower-level categories respectively. We underwent several iterations to combine and condense similar labels, until no new modifications took place.

**Figure 3:**
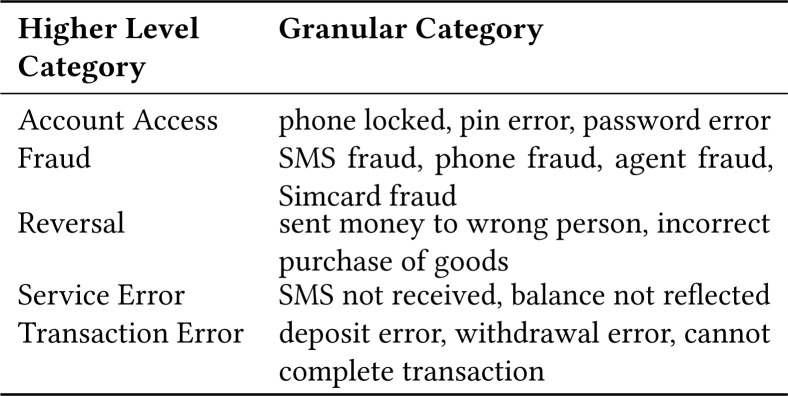
Examples of higher and lower-level labels

## 5 FINDINGS

### 5.1 Customer Support on Twitter

Some operators even had a dedicated Twitter account for customer support queries (e.g., SafariCom used @safaricom_care and @safaricom). A typical Twitter discourse begins with the customer tweeting about an issue to the support Twitter handle. Some customers’ tweets went unanswered; some tweets received just one response from support (either addressing the concern or redirecting the customer to a specific helpline); and some tweets lead to a back-and-forth conversation. When requesting more information from customers, support would ask them to share information via a Twitter direct message, but sometimes customers shared their personal information publicly on Twitter. When an issue is addressed, support would usually leave a tweet in the conversation confirming resolution of the issue or indicating that an action was taken, which also serves to promote the support as responsive and effective at handling customer issues.

### 5.2 Issues Raised on Twitter

**Figure 4:**
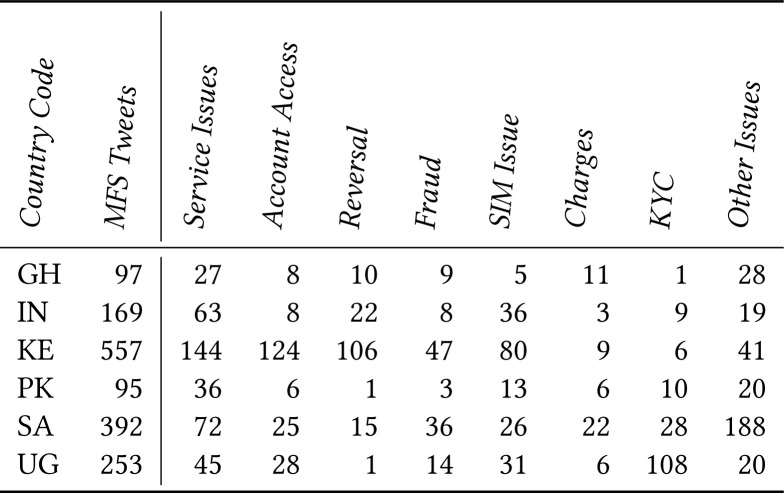
Distribution of MFS related tweets in our manually coded dataset of 9,000 tweets (1,500 tweets per country)

Figure 4 shows the common issues we identified in our dataset and the number of tweets for each issue; we now discuss these issues.

#### 5.2.1 Service and Account Access Issues

We saw several tweets about difficulties users face when using the smartphone app, SIM Toolkit App [9], or the USSD interface, include missing buttons (*“what’s up with the MySafcomApp; I can’t use it coz I can’t submit the service pin. No OK button?”*), missing menu options (*“@JazzCash this app main function is missing and fund transfer not possible in this app”*), and non-responsive interfaces (*“@MobiKwikSWAT Pl check as to why one is unable to pay thru mobi wallet on hs18 app due to some error 404.”*). Users expected to receive the payment summary immediately and would tweet if the summary did not arrive as expected. In some cases users reported urgency, as the transactions were payments for services such as electricity and telephone, and the users were worried about the services getting disconnected because of delayed payment.

The challenges that users faced around account access were primarily due to users forgetting their mobile money PIN, trying multiple times, and locking their mobile money account after exceeding the allowed retries. To unlock their mobile money account, users need a Personal Unblocking Key (PUK number), which they enter in their phone. (*“@AIRTEL_KE my simcard has blocked and I don’t have puk code”*). To get the PUK number, users tweet operators, who ask users for verification details via Twitter’s direct message to provide the same.

#### 5.2.2 Transactions Reversal

Users often sent money to an incorrect number (*“@MTNGhana Please Is there a way one can use to retrieve money sent to a wrong number”*), paid the wrong merchant or business (*“I paid some sh1000 to the wrong business no.can u plz reverse.”*), paid for a wrong service (*“I bought airtime instead of making a withdrawal”*), went to a wrong agent (e.g., one agent tweeted *“Kindly help the customer to reverse has withdraw from wrong agent”*) or entered a wrong number (e.g., *“plz reverse. I sent to 2027805-01 instead of 2720805-01”*). We found that transaction reversal requests were resolved quickly (e.g., within hours or days) when the transaction was between customers of a service provided by the same mobile money operator, but if the transaction involved a third party, resolving that issue took several days. To process reversal requests, most operators ask customers to verify their personal details and also ask the reason for reversal.

#### 5.2.3 Fraud

When customers encounter fraud, they share details about the caller and the conversation. For example, one user reported that the caller *“asked Airtel money & Mpesa registrations. Also offering 50K”*. Most of the fraud reported by users is social engineering fraud, where fraudsters pose as customer care employee and engage customers in conversation and try to extract customers’ personal identification information. Some users also reported fraudsters using threatening tone to divulge details. Unauthorized transaction was a common theme among fraud tweets in South Africa. Users reported their card being used for online transactions even though they did not lose their card or they do not remember sharing their card PIN with anyone. Most tweets about fraud were reports of fraud attempts and occurrences to providers, in which the user expected the provider to investigate and block the fraudster’s number.

#### 5.2.4 Additional Issues

In addition to the three major themes, there were minor themes that emerged and those are discussed below:

**Unexplained charges**. Tweets regarding unexplained charges highlight the issue of lack of transparency in fees associated with mobile money services. Users wanted clarification on why they had been charged certain fees or why their balance was not what they expected, like the following user.

**KYC**. Customers have to provide identification documents to create a new mobile money account or continue using their account; failure to provide the documents results in account suspension or reduced transaction and balance limits on the account. Most of the tweets in our data about KYC issues were from Uganda. Many users had trouble successfully completing their registration or had to face account suspension because some checks in their documentation failed. Also, many users did not have national ID and they would tweet seeking assistance.

**Query**. Many customers, agents, and merchants used Twitter to ask customer care for information related to various issues including commission structure for an M-Pesa agent, specific helpline numbers, how to replace a SIM while retaining mobile money account, how to access account statement.

### 5.3 Inter-Country Comparison

**Ghana, Kenya and Uganda**. We found that the mobile money ecosystem in Kenya is well developed. Users can transact money, pay bills, get insured and even apply for loans. There were also 2% Latin Swahili tweets from our sample. In comparison, the mobile money ecosystem is growing rapidly in Ghana and it is relatively new in Uganda. SIM issues were quite common in these three countries since the ecosystem is MNO led. Emerging themes became apparent such as the mandatory law in Uganda to associate the National ID number with the SIM card, rendering all unverified simcards as invalid.

**India**. Demonetization in India led to an increase in digital wallet accounts [8]. There are a few MNO led initiatives for mobile money, but the market is dominated by mobile wallet services. In India, mobile wallet companies also serve as e-commerce providers. The majority of tweets referred to defective products or delayed deliveries, however, we do not consider these tweets as MFS related. 3% of tweets in our sample were in Latin Hindi.

**Pakistan**. The MFS ecosystem in Pakistan is a hybrid of MNO led initiatives and digital wallet companies, but the majority of users use the former. Since mobile money is relatively new in Pakistan, users would ask questions and interact with customer care. 10% from our sample were in Latin and Persian Urdu.

**South Africa**. MNO led services were discontinued in 2016 and hence people use mobile banking applications in South Africa. [36]. Most tweets were about customer service complaints, Users who were not satisfied when they had called or visited a physical branch would tweet their annoyance.

## 6 DISCUSSION

### 6.1 Comparison with Other Studies

Primarily we compare our findings with the MFS challenges outlined in Section 3. Overall, the main challenges that emerged from our analysis (service error, incorrect transaction, and fraud) match with the challenges identified by prior work, and our findings complement prior studies by identifying the nuances around these high-level challenges. The differences in the findings, we believe, are due to the differences in the underlying demographics of the users: Twitter users are primarily urban, whereas FII respondents could be either urban or rural. Other differences between previous findings and ours are mainly related to usability and agent issues.

### 6.2 Twitter Analysis

Twitter provides an opportunity to learn how users tweet and interact with customer care to get issues resolved. The process for addressing problems, such as verification, is through DM or a help line, but associated information, such as persistence of customers, is exposed. Other behaviors, such as tweeting on behalf of relatives or creating Twitter accounts just for reaching the customer support are also observed. But, there is bias in Twitter data as well. Twitter users are heavily urban and middle-class [35] and not representative of the population as a whole. It is probably the case that MFS challenges detected in Twitter data are relevant across the population. Another bias is that Twitter users are usually smartphone users. Also majority tweets were from male users in South Asia, with a slightly higher percentage of African female users. Also twitter usage and complaint resolution could vary across countries.

### 6.3 Future Work

**Cross Cultural Studies**. One limitation of this work is that we excluded countries where English was not the dominant twitter language. A natural extension of this work would be to consider those countries along with some countries in Francophone Africa, Latin America, and additional countries such as Indonesia.

**Quantitative Studies**. While this work allows us to identify important issues, our analysis does not give us a quantitative understanding of MFS challenges. We would like to develop an assessment of challenges that are robust across countries and MFS providers, and can also evaluate trends over time.

**Improving MFS Technology**. A question is whether this analysis can be utilized to help address the problems described. The numerous requests for transaction reversals suggest that there are challenges associated with the menu based user interfaces, and details in the requests show that there is a variety of different mistakes that can be made. Another area of research could be to mitigate fraud. One could measure the rates of fraud reports, develop a fraud classification system and develop early warnings of new scams as they spread.

## 7 CONCLUSION

We conducted an in-depth qualitative analysis of 9,000 mobile money complaint tweets from customers in six countries. Our analysis identified seven MFS issues and highlighted the details around these issues. Our findings also illuminated how MFS services are used differently in countries and how the nature of these challenges vary. We compared our results with prior studies that identified MFS challenges, and saw that our findings match and *augment* prior studies with greater nuance. Thus, we find Twitter as a rich data source that can provide insights about how people use mobile money and the challenges they face.
